# Characterization of Bacteriophage vB-EcoS-95, Isolated From Urban Sewage and Revealing Extremely Rapid Lytic Development

**DOI:** 10.3389/fmicb.2018.03326

**Published:** 2019-01-15

**Authors:** Gracja Topka, Sylwia Bloch, Bożena Nejman-Faleńczyk, Tomasz Gąsior, Agata Jurczak-Kurek, Agnieszka Necel, Aleksandra Dydecka, Malwina Richert, Grzegorz Węgrzyn, Alicja Węgrzyn

**Affiliations:** ^1^Department of Molecular Biology University of Gdańsk, Gdańsk, Poland; ^2^Laboratory of Molecular Biology, Institute of Biochemistry and Biophysics Polish Academy of Sciences, Gdańsk, Poland; ^3^Department of Molecular Evolution University of Gdańsk, Gdańsk, Poland; ^4^Laboratory of Electron Microscopy University of Gdańsk, Gdańsk, Poland

**Keywords:** bacteriophage, coliphage, lytic development, genomic analysis, biofilm

## Abstract

Morphological, biological, and genetic characteristics of a virulent *Siphoviridae* phage, named vB-EcoS-95, is reported. This phage was isolated from urban sewage. It was found to infect some *Escherichia coli* strains giving clear plaques. The genome of this phage is composed of 50,910 bp and contains 89 ORFs. Importantly, none of the predicted ORFs shows any similarity with known pathogenic factors that would prevent its use in medicine. Genome sequence analysis of vB-EcoS-95 revealed 74% similarity to genomic sequence of *Shigella* phage pSf-1. Compared to pSf-1, phage vb-EcoS-95 does not infect *Shigella* strains and has an efficient bacteriolytic activity against some *E. coli* strains. One-step growth analysis revealed that this phage has a very short latent period (4 min), and average burst size of 115 plaque forming units per cell, which points to its high infectivity of host cells and strong lytic activity. The bacteriolytic effect of vB-EcoS-95 was tested also on biofilm-producing strains. These results indicate that vB-EcoS-95 is a newly discovered *E. coli* phage that may be potentially used to control the formation of biofilms.

## Introduction

Bacteriophages (or phages), i.e., viruses infecting bacterial cells, represent the most abundant biological creatures on Earth (Clokie et al., 2011). Number of phage virions is estimated at the level of 10^31^ (Weitz et al., 2012). These viruses have been discovered over 100 years ago (Twort, 1915; summarized by Wegrzyn and Wegrzyn, 2015). Some of them have played an extremely important role in development of molecular biology, serving as model organisms in studies on basic cellular processes, including gene expression, replication of genetic material, developmental regulation, environmental stress responses, and others (Wegrzyn and Wegrzyn, 2005; Krisch and Comeau, 2008; Casjens and Hendrix, 2015). Moreover, bacteriophages have been extensively used in genetic engineering and biotechnology, serving as cloning vectors and providing genetic elements used in construction of strictly regulated gene expression systems, phage display systems, and others (Onodera, 2010).

The idea of the use of bacteriophages to combat bacterial infections appeared shortly after discovery of these viruses (for a review, see Wegrzyn and Wegrzyn, 2015). Early experimental phage therapy was promising, however, discovery of penicillin led to the believe that every bacterial infection can be eliminated by the use of antibiotics, and the idea of phage therapy has been largely abandoned (Kakasis and Panitsa, 2019). Recently, the return to the use of bacteriophages to combat bacterial infections is evident. In the era of the antibiotic crisis, phage therapy appears to be one of alternatives to treat infections with bacterial strains resistant to most, if not all, known antibiotics (Kutter et al., 2015; Domingo-Calap et al., 2016; Gorski et al., 2018a). Phage therapy is based on the simple expectation that viruses which can destroy bacterial cells can be an excellent tool to eliminate specific infection agents in human (or animal) body. The major advantage of this kind of therapy is its specificity [bacteriophages are usually specific to single bacterial species, and often to particular strain(s)], auto-control (phages should propagate only if their host bacteria are available), and safety (phages do not infect eukaryotic cells) (Górski et al., 2018b). However, some potential problems must also be considered, including possibility to lysogenize host cells by a phage instead of lysing it (thus, temperate phages should not be used in phage therapy), the presence of toxin genes in genomes of some bacteriophages (such phages must be avoided in phage therapy), and possibility of development of phage resistance by bacteria (Kakasis and Panitsa, 2019). Nevertheless, antibacterial activities of bacteriophages are so attractive that the use of these viruses has been extended to food protection (Gutiérrez et al., 2016), agriculture and industry (Domingo-Calap and Delgado-Martínez, 2018), i.e., in every area of human action where bacteria may cause unwanted effects.

In the light of the extremely large population of bacteriophages (10^31^ virions on Earth, as mentioned above), a huge variability of these viruses (Hatful, 2015) is perhaps not a surprise. What is the surprise, is the relatively poor knowledge on this variability and relatively low number of characterized bacteriophages relative to other organisms (discussed by Jurczak-Kurek et al., 2016). Our previous studies indicated that even if bacteriophages are isolated from a single habitat, their diversity is huge (Jurczak-Kurek et al., 2016). Moreover, many phages isolated from environmental samples reveal properties that are very promising from the point of view of their applications in biotechnology or medicine (Jurczak-Kurek et al., 2016).

In this report, we describe detailed characterization of bacteriophage vB-EcoS-95, isolated from urban sewage, including virion morphology, host range, developmental kinetics, and genome analysis. Specific features of this bacteriophage, infecting *Escherichia coli* strains, particularly an extremely short latent period and ability to destroy bacterial biofilm, suggest that it can be used in further studies on development of novel biotechnological tools and/or its use in food protection/medicine.

## Materials and Methods

### Bacterial Strains and Growth Conditions

Bacterial strains used in this study are listed in Table 1. *E. coli* strains and *Pseudomonas* bacteria were cultured in liquid Luria–Bertani broth (LB) or plated on solid LB medium with 1.5% agar (LA medium; Lab Empire). For *Enterococcus* and *Shigella*, special Tryptic Soy Broth (TSB) and Tryptic Soy Agar (TSA) were used (BTL). The liquid cultures were grown with aeration at 37°C in a shaking incubator (200 rpm; Eppendorf). The plates with solid medium were incubated at 37°C for 24 h. The phage infection processes were studied at 37°C, under aerobic conditions in a shaking incubator (200 rpm; Eppendorf). Biofilm studies were performed by using *E. coli* MG1655 strain bearing pUC18 plasmid and F′ plasmid from *E. coli* ER2738 strain. These bacteria were grown at 37°C without shaking in 12-well polystyrene plate with M9 medium containing 0.2% glucose (POCH).

**Table 1:** Bacterial strains and plasmid used in the study.

**Bacterial strain**	**Source or references**	**Relevant genotype or other characteristics**
***E. coli*** **LABORATORY STRAINS**		
MG1655	Jensen, 1993	F- λ–* ilvG rfb-50 rph-1*
MG1655 producing biofilm	This study	[pUC18] F^+^ Amp^R^, Tet^R^
C600 Hfr3000	Appleyard, 1954 Bachmann, 1972	F- *tonA21 thi-1 thr-1 leuB6 lacY1 glnV44 rfbC1 fhuA1 λ –*
Tap90	Patterson and Dean, 1987	F- *recD1903::mini-tet supE44 supF58 lacY1 pro leuB6 hsdR rpsL tonA1 thi-1* lambda-
ER2738 MC1061 DH5α ATCC® 25922™	Gough and Murray, 1983 Casadaban and Cohen, 1980 Grant et al., 1990 ATCC: The Global Bioresource Center	F'*proA^+^B^+^ lacI^*q*^ Δ(lacZ)M15 zzf::Tn*10(Tet^R^)*/ fhuA2 glnV Δ(lac-proAB) thi-1 Δ(hsdS-mcrB)5 F Δ(ara-leu)7697 [araD139]B/r Δ(codB-lacI)3 galK16 galE15 λ- e14- mcrA0 relA1 rpsL150(strR) spoT1 mcrB1 hsdR2(r-m+) F- endA1 glnV44 thi-1 recA1 gyrA96 deoR nupG Φ80dlacZΔM15 Δ(lacZYA-argF)U169, hsdR17(rK- mK+), λ-* Serotype O6
***E. coli*** **CLINICAL STRAINS**		
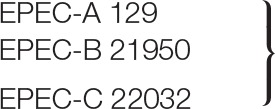	Specialist Hospital of St. Wojciech in Gdansk (Poland) Griffin et al., 1988 Beutin et al., 1989 Beutin et al., 1989; Perna et al., 2001 Specialist Hospital of St. Wojciech in Gdansk (Poland)	Stool isolate; EspA Stool isolate; EspB Stool isolate; EspC
EHEC O157:H7 ST2-8624		Stool isolate; Stx1 and Stx2
EHEC O157:H7 CB571		Stool isolate; Stx1 and Stx2
EHEC O157:H7 EDL933		Stool isolate; Stx1 and Stx2
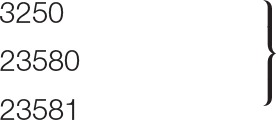		Stool isolate
		Stool isolate
		Stool isolate
**OTHERS STRAINS**		
*Enterococcus faecalis* 230	Department of Water and Waste-Water Technology of Gdansk University of Technology	Urban sewage isolate
*Pseudomonas aeruginosa* 575/2003	National Medicines Institute in Warsaw (Poland)	Decubitus ulcer isolate
*Pseudomonas aeruginosa* 1947/2003	National Medicines Institute in Warsaw (Poland)	Wound isolate
*Salmonella enterica* Anatum	National Salmonella Center at Medical University of Gdansk (Poland) National Salmonella Center at Medical University of Gdansk (Poland)	-
*Salmonella enterica* Heidelberg		-
*Salmonella enterica* Panama	National Salmonella Center at Medical University of Gdansk (Poland)	-
*Salmonella enterica* Reading	National Salmonella Center at Medical University of Gdansk (Poland)	-
*Shigella flexneri* 12022	ATCC: The Global Bioresource Center	-
*Staphylococcus sciuri* IO	Institute of Oceanology of Polish Academy of Sciences in Sopot (Poland)	Urban sewage isolate
**PLASMID**		
**pUC18**	Thermo Fisher Scientific	ori pMB1 (pBR322 derivative), *bla*, Amp^R^

*EHEC, Enterohemorrhagic E. coli; EPEC, Enteropathogenic E. coli; Stx, Shiga toxin; Esp, E. coli secretion protein*.

### Isolation of Bacteriophage vB_Ecos-95 From Urban Sewage

Bacteriophage vB_EcoS-95 was isolated from urban sewages, according to protocols described by Jurczak-Kurek et al. (2016). Water samples were collected from Gdansk Wastewater Treatment Plant in Poland. In the first stage of the procedure, 10 ml of the sewage sample were mixed with 1 ml of the *E. coli* MG1655 overnight culture and cultivated for a few hours at 37°C with shaking. The obtained phage lysate was clarified by centrifugation (10,000 × g, 30 min, 4°C) and extracted several times with chloroform (POCH). To obtain visible plaques, formed by phages, 50 μl of the supernatant were added to 2 ml of the overnight host bacterial culture and plated using the agar double layer method (Sambrook and Russell, 2001). Next day, a single plaque was scraped with a sterile bacteriological loop and transferred to a flask with mid-log phase *E. coli* MG1655 strain. After the lysis of bacterial culture occurred, chloroform extraction was carried out and phages were re-plated on a lawn of *E. coli* MG1655 strain. Serial dilutions of the phage lysate in TM buffer (10 mM Tris-HCl, 10 mM MgSO_4_; pH 7.2**)** were prepared. Then, appropriate volume of each dilution was spotted onto double agar layer to obtain single phage plaques that were propagated three times by this method to obtain purified vB_EcoS-95 lysate. For further analysis, the required amount of vB_EcoS-95 lysate was prepared by adding phage particles to the exponentially growing *E. coli* MG1655 bacteria which were then cultivated with shaking at 37°C (200 rpm; Eppendorf) until lysis occurred. In the next step, phage particles, released during lysis, were purified by centrifugation and extracted with chloroform. Phage activity was determined using a conventional double-layer agar technique (Sambrook and Russell, 2001).

### Host Range Determination

The phage host range spectrum was determined using different bacterial strains which are listed in Table 2. The spot lysis assay was prepared onto a double layer agar plate, following the protocol described by Jurczak-Kurek et al. (2016). Briefly, standard Petri dishes were filled with 25 ml of LA (*E. coli* and *Pseudomonas* strains) or TSA (*Enterococcus* and *Shigella* strains) medium. Then, 3 ml of LB (*E. coli* and *Pseudomonas* strains) or TSB (*Enterococcus* and *Shigella* strains) medium, supplemented with 0.7% agar/0.4% agarose, were mixed with 1 ml of the overnight bacterial culture and poured onto LA or TSA bottom agar, respectively. Ten-fold dilutions of vB_EcoS-95 lysate were prepared in TM buffer (10 mM Tris-HCl, 10 mM MgSO_4_; pH 7.2) and spotted onto the surface of the double-layer agar plates with a tested host. Plates were incubated at 37°C for 24 h. Productive interaction of the bacteriophage with the host bacterium was revealed as the presence of plaque formation. According to the degree of clarity, the observed results were differentiated into three classes: (++) clear plaques, (+) turbid plaques, and (−) no plaques.

**Table 2:** Host spectrum of bacteriophage vB_EcoS-95.

**Bacterial strain**	**Plaques**
*Escherichia coli* MG1655	++
*Escherichia coli* C600	++
*Escherichia coli* TAP90	++
*Escherichia coli* Hfr3000	++
*Escherichia coli* MC1061	++
*Escherichia coli* DH5α	++
*Escherichia coli* ATCC 25922	−
*Shigella flexneri* (12022)	−
*Salmonella enterica* (Anatum)	−
*Salmonella enterica* (Heidelberg)	−
*Salmonella enterica* (Reading)	−
*Salmonella enterica (*Panama)	−
*Pseudomonas aeruginosa* (575)	−
*Pseudomonas aeruginosa* (1947)	−
*Enterococcus faecelis* (230)	−
*Staphylococcus sciuri*	−
*Escherichia coli* EPEC-A	−
*Escherichia coli* EPEC-B	−
*Escherichia coli* EPEC-C	−
*Escherichia coli* EHEC O157:H7 *E. coli* EHEC O157:H7 ST2-8624 *E. coli* EHEC O157:H7 CB571 *E. coli* EHEC O157:H7 EDL933	−−−−
Clinical isolate of *E. coli* 3250	++
Clinical isolate of *E. coli* 23580	++
Clinical isolate of *E. coli* 23581	+

*Symbols: (++) clear plaques, (+) turbid plaques or (−) no plaques after infection with phage*.

### Microscopic Analyses

Virions were purified from the phage lysate (2.1 × 10^10^ PFU/ml) obtained after infection of the host strain *E. coli* C600 with phage vB_EcoS-95. Purification was performed using cesium chloride density gradient centrifugation method, described by Sambrook and Russell (2001). Electron microscopic analyses of phage particles were performed employing the Philips CM 100 electron microscope (Philips, Eindhoven, The Netherlands), by using negative staining with uranyl acetate (Czajkowski et al., 2015). Dimensions of virions were measured on micrographs at magnification of 39,000 times, with analySIS Pro (iTEM) software. The Tomocube holographic 3D microscope (Perlan Technologies, Poland) was used to evaluate the structure of *E. coli* biofilms after 4 h incubation with phage vB_EcoS-95 lysate (2.1 × 10^10^ PFU/ml).

### Determination of Plaque Morphology

Plaque morphology of phage vB_EcoS-95 was tested on the *E. coli* C600 strain. To determine plaque size, serial dilutions of phage lysate in TM Buffer (10 mM Tris-HCl, 10 mM MgSO_4_) were prepared. Next, 1 ml of the host strain culture was mixed with 10 μl of each dilution of phage lysate and added to 3 ml of top LB with 0.7% agar. The mixture was spread onto LA plate. Diameters of plaques were measured manually and pictures were taken using the digital scanner HP Scanjet G4050 and assigned software.

### The Influence of the External Factors on Phage Particles Stability

Phage stability tests were performed according to protocols described by Jurczak-Kurek et al. (2016). To determine the sensitivity of phage lysate, the following external factors were tested: temperature (−20, 20, 30, 37, 40, 62, and 95°C), pH (2, 4, 10, and 12), organic solvents (ethanol, chloroform, DMSO, and acetone), and detergents (SDS, sarkosyl, and CTAB). The survival of phages during osmotic shock conditions was also analyzed.

### Efficiency of Bacteriophage Adsorption

Bacteria were grown in LB medium at 37°C to OD_600_ = 0.3. Samples of 6 ml were centrifuged and pellets were washed with 1 ml of 0.85% NaCl (Chempur). After centrifugation, each pellet was suspended in 1.3 ml of LB medium and incubated at 37°C for 15 min. Then, bacteriophage lysate was added to m.o.i. = 0.1. During the incubation, samples were withdrawn at indicated times and centrifuged (6000 × g for 1 min at room temperature). In the next step, the supernatants were titrated. Plates were incubated at 37 °C overnight. A sample withdrawn immediately after addition of phage lysate to the bacterial host strain (time zero) was considered as 100% non-adsorbed phages. Other values were calculated relative to this value.

### One-Step Growth Analysis

Intracellular lytic development of phage vB_EcoS-95 was studied in one-step growth experiment, according to the procedure described by Bloch et al. (2013), with minor modifications. Briefly, *E. coli* MG1655 host cells were grown with shaking in LB medium at 37°C to OD_600_ = 0.2. Then, 10 ml of culture sample were harvested by centrifugation (4,000 × g, 10 min, 4°C). The supernatant was discarded, and the pellet was resuspended in 1 ml of fresh LB medium with 3 mM sodium azide (Sigma-Aldrich). Following 5-min incubation at 37°C, bacteriophage vB_EcoS-95 was added to *E. coli* MG1655 to m.o.i. = 0.01. After 10 min incubation at 37°C, non-adsorbed phage particles were removed by three times washing with 1 ml of LB medium containing 3 mM sodium azide (4,000 × g, 10 min, 4°C). In the next step, 25 μl of the suspension was added to 25 ml of LB medium (time 0), and aerated in an incubator shaker at 37°C. The number of phage-infected bacterial cells was determined at time 1 min after infection by mixing 5 μl of the culture sample with 0.995 ml of an overnight *E. coli* MG1655 culture and 2 ml of top agar (LB with 0.7% agar), prewarmed to 45°C. Next, the mixture was poured onto LA plate (infected bacteria were named “infection centers” because they were sources of new phage particles, which were released from host cells during one lytic cycle, and following infection of neighboring cells could form plaques). Two sets of samples were collected every 1 min during first 10 min, and then every 2.5 min. The samples were serially diluted (10-fold each) in TM buffer (10 mM Tris-HCl, 10 mM MgSO_4_) and titrated under permissive conditions. Before the titration, the second set of samples was treated with 1% chloroform (final concentration) to release the intracellular phage particles to determine the eclipse period. Based on the number of PFU/ml, the latent period and burst size were determined (the burst size was estimated as the ratio of the phage titer to the titer of infection centers).

### Lysis Profile Assay

Host culture was grown to OD_600_ = 0.2 at 37°C. Phage lysate was add to m.o.i. = 0.05 to the flask. Bacteria were incubated with shaking at 37°C, and their density was monitored by OD_600_ measurement in the same time intervals (every 5 min). During this experiment, survival of host bacteria after phage infection (CFU/ml) and phage titer (PFU/ml) were also analyzed. To estimate the number of surviving cells after the vB_EcoS-95 infection, 100 μl samples were collected at times indicated above, and their serial 10-fold dilutions were prepared in LB. Forty μl of each dilution were spread onto LB agar plates. The number of viable bacterial cells was calculated on the basis of counted colonies. To determine the number of phages per ml, samples were taken every 5 min and their serial 10-fold dilutions were prepared in TM buffer. Next, 2.5 μl of each dilution were spotted onto double agar layer, and then plates were incubated overnight at 37°C. The phage titer was calculated on the basis of counted plaques.

### Development of Bacterial Resistance

The emergence of bacterial resistance was measured for the combination of phage vB_EcoS-95/antibiotic rifampicin (Lab Empire) and the *E. coli* MG1655 strain. Host bacteria were cultured to OD_600_ = 0.2 at 37°C. Next, the culture was divided into three aliquots. Phage lysate was added to one of the flasks to m.o.i. of 0.01. The second one was treated with rifampicin to a final concentration of 25 μg/ml. The last one was a control. The cultivation was continued at 37°C. After 3 h of shaking, 10 μl of each samples were withdrawn. Serial dilutions in LB medium were prepared and 90 μl of each dilution was spread on LB agar plates. After overnight incubation at 37°C, percentage of surviving *E. coli* bacteria after phage infection or rifampicin treatment was calculated relative to bacterial control. To estimate the percent of bacterial colonies resistant to phage vB_EcoS-95 infection, 300 colonies were passaged in each well of a 24-well plate with 2 ml of LB medium and shaken at 37°C to OD_600_ = 0.1. Next, the phage lysate was added to each well to m.o.i. of 1. The lack of bacterial cell lysis indicated the emergence of bacterial resistance to phage vB_EcoS-95 infection. To calculate the percent of bacteria resistant to tested antibiotic, the same number of bacterial colonies was passaged onto LB agar plates supplemented with rifampicin to final concentration of 25 μg/ml, and incubated for 24 h at 37°C.

### Quantification of Biofilm Density After Phage Infection

Biofilm cell density after phage infection was determined according to the protocols described by Sung et al. (2006) and Vianney et al. (2005), with some modifications. For biofilm cell culture, *E. coli* MG1655/pUC18 F′ strain was grown at 37°C in 12-well polystyrene plate with M9 medium containing 0.2% glucose. After 48 h of growth, the liquid medium containing planktonic cells was removed. The surface-attached cells in the biofilm were washed with 1 ml of 1 × PBS. In the next step, phage lysate was added to each well, except for controls, to the final titer of 10^2^, 10^4^, 10^5^, 10^7^ or 10^10^ PFU/well, and then plates were incubated at 37°C for 4 h. In the case of control wells, the medium was added instead of phage lysate. Fallowing the incubation, phage lysate was removed and surface-attached cells were resuspended in 1 ml of 1 × PBS. The biomasses of bacterial biofilm were estimated by measuring the optical density (OD) with a plate reader (EnSpire Multimode Plate Reader) at wavelength 600 nm. In order to take photos of biofilms for densitometry analyses, phage lysate was removed and surface-attached cells were dried at 37°C for 15 min. Biofilm area intensities were quantified from the performed images by densitometry, using QuantityOne software (Bio Rad®).

### Assessment of Biofilm Biomass by Crystal Violet (CV) Staining

Biofilm cells were prepared according to the procedure described above. After the incubation with phage lysate (added to final titer of 10^2^,10^4^,10^5^,10^7^, or 10^10^ PFU/well), the liquid medium containing planktonic bacteria was carefully removed and surface-attached cells were treated with 0.5 ml of 0.1% crystal violet (Sigma-Aldrich). Plates were incubated in the dark for 30 min at room temperature. In the next step, crystal violet was carefully removed and biofilms were washed 5 times with 1 ml of 1 × PBS. Then, biofilms were fixed by incubating the plates at 60°C for 30 min. Following the incubation, crystal violet was dissolved by the addition of 1 ml of 96% ethanol. To determine the biomass of bacterial biofilm, absorbance was measured in a plate reader at 570 nm (EnSpire Multimode Plate Reader). Biofilm area after fixation was also photographed to visualize the differences in biofilm biomass after phage lysate treatment.

### Assessment of Metabolic Activity of Biofilm Cells by Resazurin

Biofilms were prepared in the same way as described above. Briefly, after incubation with phage lysate (added to final titer of 10^2^,10^4^,10^5^,10^7^,10^10^ PFU/well), the liquid medium was removed. The biofilms were suspended in 1 ml of 1 × PBS. In the next step, resazurin was diluted in PBS buffer and added to each well to a final concentration of 6 μg/ml. Plates were gently shaken and incubated for 150 min at room temperature. The fluorescence of the produced resorufin (λ_exc_ = 570 and λ_em_ = 590 nm) was measured every 15 min in a plate reader (EnSpire Multimode Plate Reader). Results are presented in Fluorescent Units (FU).

### Phage DNA Isolation

The phage lysate was treated with DNase I (1 U/μl; Thermo Fisher Scientific) and RNase A (5 ug/μl; Thermo Fisher Scientific) to degrade bacterial nucleic acids. To digest the exogenous DNA and RNA, the mixture was incubated for 30 min at 37°C. Then, DNase I and RNase A were inactivated by heating to 95°C and the genomic DNA of phage vB_EcoS-95 was isolated with a MasterPure™ Complete DNA and RNA Purification Kit (Epicenter). The DNA concentration was determined spectrophotometrically at 260 nm.

### Sequencing of vB_EcoS-95 Genome

Phage genomes were sequenced in the Genomed company involving Next Generation Sequencing (NGS) and MiSeq (Illumina) genome sequencer. Assembly of the sequences was accomplished by the experts from the Genomed bioinformatics group. The quality of vB_EcoS-95 reads was controlled using FastQC (https://www.bioinformatics.babraham.ac.uk/projects/fastqc/), with following parameters: -*q* = 20 and -*m* = 36. The raw data (792,164 raw reads) were filtered using a Cutadapt program (http://code.google.com/p/cutadapt/) to remove the adapters, N bases, and low-quality reads. *De novo* assembly (99.97% of raw reads) was conducted using CLC Genomics Workbench. Finally, the assembly generated a single contig, corresponding to the entire phage vB-EcoS-95 genome with an average coverage of 2,629 x. Additionally, the obtained sequencing results, were analyzed for any errors in contigs assembly using following programs: BLAST (http://blast.ncbi.nlm.nih.gov/Blast.cgi), Progressive MAUVE (http://darlinglab.org/mauve/mauve.html) and Serial Cloner software (http://serialbasics.free.fr/Serial_Cloner.html).

### Bioinformatic Analysis of vB_EcoS-95 Genome

The fully assembled genome of phage vB_EcoS-9 was annotated using myRAST software (Caldeira and Peabody, 2007) and UGENE bioinformatics software (http://ugene.net/) (Essoh et al., 2015). The identification of the putative protein-coding genes was based on the presence of a plausible ribosome binding site, and both the start and stop codons. Furthermore, genome annotation was verified and curated by BLAST analysis, HMMER software (http://www.hmmer.org/), Phobious webserver (http://phobius.binf.ku.dk/) and TMHMM program (http://www.cbs.dtu.dk/services/TMHMM/). Circular map of vB_EcoS-95 phage genome was generated using BLAST Ring Image Generator (BRIG) platform (https://sourceforge.net/projects/brig/) and CGView was used to perform GC skew and GC content analyses (Stothard and Wishart, 2005). A linear visualization of alignments of vB_EcoS-95 and pSf-1 (accession number KC710998) genomes was generated by Easyfig program (http://mjsull.github.io/Easyfig/files.html). The Neural Network Promoter Prediction NNPP method (http://www.fruitfly.org/seq_tools/promoter.html) was used to find phage-specific promoters in a DNA sequence. The positions of Rho-independent transcriptional terminators were determined using FindTerm tool (http://www.softberry.com/berry.phtml). Finally the genome sequence of the *E. coli* phage vB_EcoS-95 with annotations was deposited in the GenBank database under the accession number MF564201.

### Construction of Phylogenetic Tree

Based on the predicted amino acid sequences, obtained from the genome analysis, phylogenetic analysis for the phage was made. To construct the phylogenetic tree, the sequence for the terminase large subunit (TerL), which is universally used genetic marker for the order *Caudovirales*, was selected. The terminase sequence of the vB_EcoS-95 phage was aligned with those of other reference bacteriophages within the order *Caudovirales*, which were collected from the NCBI database, using MUSCLE implanted in the MEGA (http://www.megasoftware.net/). A neighbor-joining phylogenetic tree for terminase large subunit amino acid sequences was constructed using the Poisson model. The robustness of the tree topology was assessed by bootstrap analyses based on 1,000 random resamplings.

### Phage Protein Extraction

All protein analyses were carried out at the Institute of Bioorganic Chemistry of Polish Academy of Sciences. In the first step, the phag**e** suspension in TM buffer (10 mM Tris-HCl, 10 mM MgSO_4_) was treated with 4 volumes of ice-cold acetone and incubated at −20°C for 30 min. Then, the sample was centrifuged (13,000 × g, 5 min, 4°C) and the supernatant was discarded. The pellet was dried in laminar flow hood at room temperature. Then, the pellet was reconstituted in 50 mM ammonium bicarbonate in the initial sample volume. The total concentration of phage proteins was measured by using BCA colorimetric assay (Thermo Fisher Scientific).

### In-Solution Tryptic Digestion

An aliquot of phage proteins was treated with 5.6 mM dithiothreitol (DTT) in 50 mM ammonium bicarbonate prior to heating at 95°C for 5 min. The sample was allowed to cool to room temperature and then was alkylated with 5 mM iodoacetamide. The mixture was incubated in the dark, at room temperature for 20 min. In the next step, phage proteins were digested with 0.2 μg of sequencing-grade trypsin (Promega). After overnight incubation at 37°C, the trypsin activity was suppressed by adding trifluoroacetic acid (TFA) to a final concentration of 0.1%. Then, the mixture was transferred to HPLC conical vial.

### Mass Spectrometry Analysis of Proteins

The analysis of phage proteins was performed by using Dionex UltiMate 3000 RSLC nanoLC System connected to QExactive Orbitrap mass spectrometer (Thermo Fisher Scientific). Peptides derived from in-solution digestion were separated on a reverse phase Acclaim PepMap RSLC nanoViper C18 column (75 μm × 25 cm, 2 μm granulation) by using acetonitrile gradient (from 4 to 60%, in 0.1% formic acid) at 30°C and a flow rate of 300 nL/min (for 185 min). Mass spectra were acquired on the Q Exactive in a data-dependent mode by using top 10 data-dependent MS/MS scans. Target value for the full scan MS spectra was set to 1e6 with a maximum injection time of 100 ms and a resolution of 70,000 at m/z 400. The 10 most intense ions charged two or more were selected with an isolation window of 2 Da and fragmented by higher energy collisional dissociation with NCE 27. The ion target value for MS/MS was set to 5e4 with a maximum injection time of 100 ms and a resolution of 17.500 at m/z 400.

### Analysis of Proteomic Data

Identification of proteins was performed by using database created from translated open reading frames that have been found in the genome of phage vB_EcoS-9 (the precision of tolerance for peptide and fragment of ion masses was 10 ppm and 0.8 DA, respectively). All raw data obtained for each dataset were imported into Proteome Discoverer 1.4 software (Thermo Scientific). Protein was considered as positively identified if at least two peptide spectral matches per protein were found by Sequest search engine, and a peptide score reached the significance threshold FDR = 0.05.

### Statistical Analyses

Statistical significances were determined using *t*-test. The significance of differences between compared experimental variants were marked by asterisks *P* < 0.05 (^*^), *P* < 0.01 (^**^) or *P* < 0.001 (^***^).

## Results

### Isolation of vB-EcoS-95 and Host Range

The vB-EcoS-95 bacteriophage was isolated as a virus infecting *E. coli* from samples of urban sewage. The isolation procedure was based on the one-host enrichment method in which a raw urban sewage sample was mixed with a culture of *E. coli* MG1655 strain to obtain the lysate of vB-EcoS-95 bacteriophage, as described in the Material and Methods section.

We found that this bacteriophage is able to efficiently infect different *E. coli* strains, including those lysogenic with various lambdoid phages. However, Shiga toxin-producing *E. coli* serotype O157:H7 was resistant to vB-EcoS-95 (Table 2). Moreover, vB-EcoS-95 is not able to infect other bacterial species, including other species from *Enterobacteriaceae*, like *Shigella flexneri* or *Salmonella enterica* (Table 2).

### Plaque and Virion Morphology

**Figure 1:**
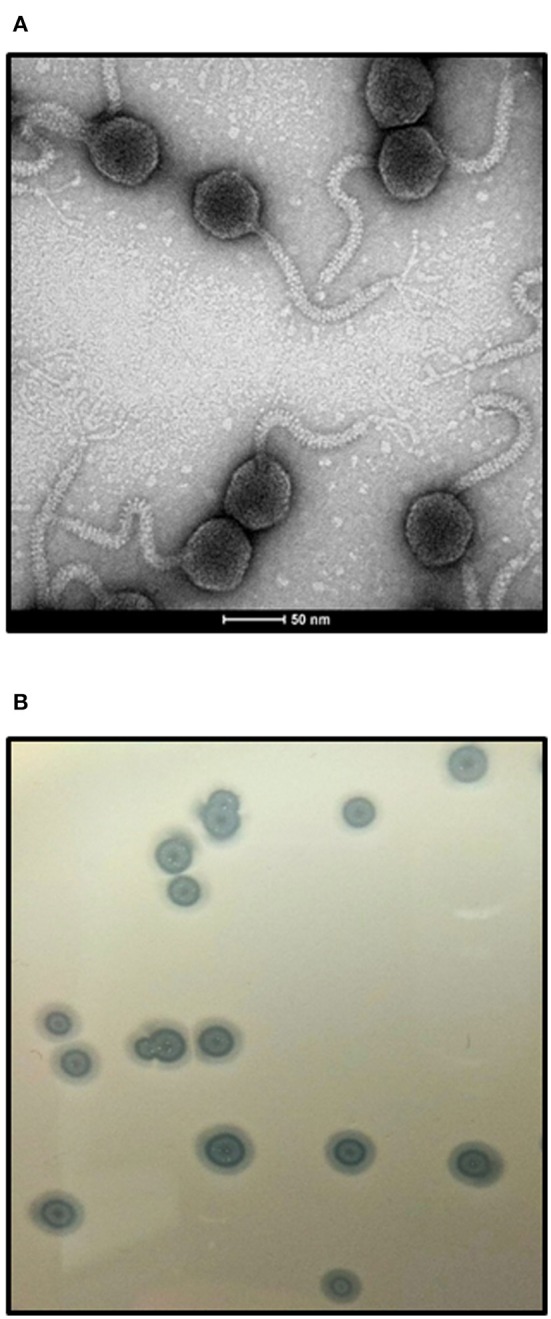
Electron micrograph of phage vB_EcoS-95 **(A)** and its plaques formed in double-layer agar plates with *E. coli* MG1655 strain **(B)**. The bar corresponds to 50 nm.

Bacteriophage vB-EcoS-95 forms clear plaques (diameter 2.5 ± 0.5 mm), with a halo, on *E. coli* lawn (Figure 1). Electron microscopic studies indicated that the virion of this phage consists of a head (diameter 53 nm), flexible, non-contractile tail (127 nm length, 10 nm width), and 3 tail fibers (about 39 nm length, 2.5 nm width). Therefore, taking into account the morphological characteristics of phage vB-EcoS-95, we conclude that this bacterial virus belongs to the *Siphoviridae* family (Table 3).

**Table 3:** Morphological characteristic of bacteriophage vB_EcoS-95.

**Phage name**	**Head lenght [nm]**	**Head diameter [nm]**	**Tail lenght [nm]**	**Tail diameter [nm]**	**Tail fiber lenght [nm]**	**Tail fiber diameter [nm]**	**Phage family**	**Plaque morphology**
vB_EcoS-95	54	53	127	10	39	2.5	Siphoviridae	Clear; ø 2−3 mm

### Sensitivity of Virions to Physical and Chemical Factors

We have tested sensitivity of vB-EcoS-95 virions to physical and chemical factors. The results, presented in Table 4, indicate that this phage is particularly sensitive to high temperature, low pH, the presence of SDS (ionic detergent) and organic solvents, like ethanol and acetone. It is, however, resistant to freezing, osmotic shock, and chloroform (Table 4).

**Table 4:** Resistance of phage virion to physical and chemical agents.

**Phage name**	**Lysis at 4°C**	**-20°C**	**20°C**	**30°C**	**37°C**	**40°C**	**62 °C**	**95°C**	**pH 2**	**pH 4**	**pH 10**	**pH 12**	**Osmotic shock**	**0.09% SDS**	**0.1% CTAB**	**0.1% Sarkosyl**	**63% Ehtanol**	**90% Acetone**	**50% DMSO**	**Chloroform**
vB_EcoS-95	+/−	92	100	82	82	44	4.1	0	0	0.43	93	0.27	86	0	67	14.7	0.8	0.3	71	87

*Comparison of effects of physical and chemical agents on phage vB_EcoS-95. Percent of surviving phages under certain conditions is shown*.

### Kinetics of Adsorption on Host Cells and Intracellular Development

**Figure 2:**
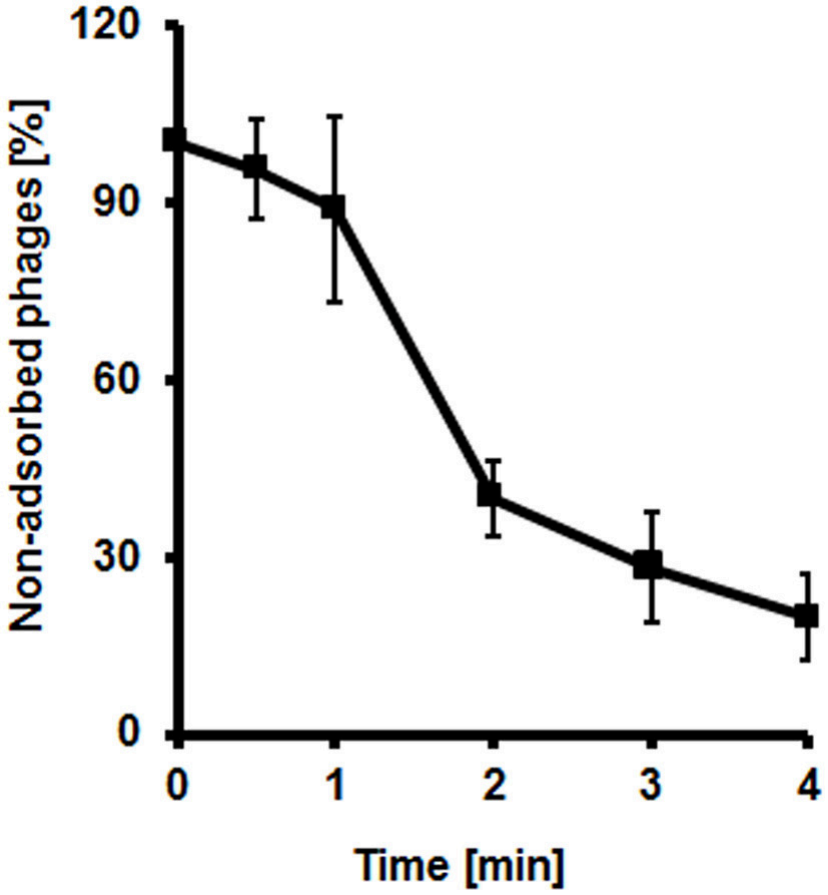
Kinetics of vB_EcoS-95 phage adsorption on host cells. Results are presented as mean values ± SD from three independent experiments.

We have determined that bacteriophage vB-EcoS-95 adsorbs rapidly on *E. coli* cells, with 50% phages already adsorbed within 2 min after mixing phage lysate with bacterial culture (Figure 2).

**Figure 3:**
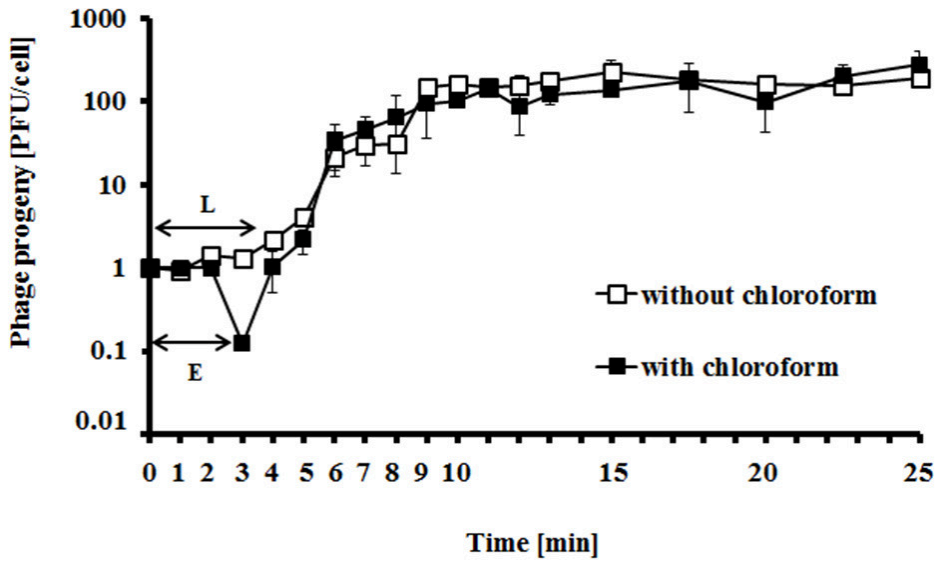
Kinetics of progeny production by vB_EcoS-95 phage in the single life cycle. Closed squares, chloroform-treated sample; open squares, non-chloroform-treated sample. E, eclipse period; L, latent period. Results are presented as mean values ± SD from three independent experiments, and shown as PFU (plaque-forming units) per 1 cell.

Intracellular phage development appeared extremely rapid, with the eclipse and latent periods 3 min and 4 min, respectively (Figure 3). The average bust size in *E. coli* grown at 37°C in LB medium has been estimated to be 115 PFU (plaque forming units) per cell, indicating efficient lytic development of the phage (Figure 3).

### Lysis Profile Assay and Development of Bacterial Resistance

**Figure 4:**
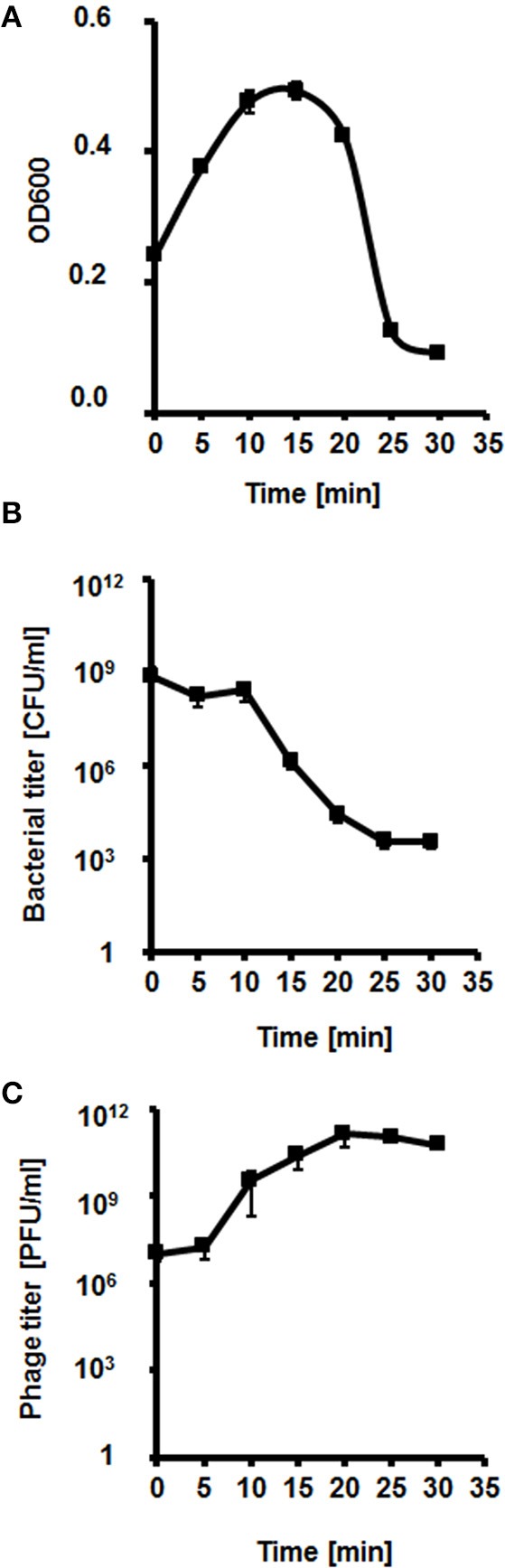
Kinetics of lytic development of bacteriophage vB_EcoS-95 in *E. coli* MG1655 bacteria. Results are shown as **(A)** bacterial culture density measured at OD_600_, **(B)** a number of surviving cells after the vB_EcoS-95 infection per 1 ml (CFU/ml), and **(C)** a number of phages per 1 ml (PFU/ml). Results are presented as mean values ± SD from three independent experiments. Please note that in some cases, the bars are smaller than sizes of symbols.

We have analyzed the lysis profile of phage vB-EcoS-95, taking account the bacterial density after addition of phage particles, the number of surviving host bacteria after phage infection, and the increase of the phage titer during this process (Figure 4). We observed that the lysis of host culture was very rapid and complete within 20–25 min. Interestingly, the emergence of resistance of *E. coli* bacteria against phage vB-EcoS-95 was similar to that against antibiotic rifampicin (Table 5). However, the percentage of surviving host cells after phage infection was considerably lower than that observed in antibiotic-treated bacteria (Table 5).

**Table 5:** Appearance of *E. coli* MG1655 resistance to phage vB_EcoS-95 and to rifampicin.

**Factor**	**Surviving bacteria (% of control) after 3 h of incubation**	**% of resistant bacteria among survivors**
Phage (m.o.i. = 0.01)	<0.01	97.22 ± 2.40
Rifampicin (25 μg/ml)	3.80 ± 1.49	99.65 ± 0.60

### Ability of vB-EcoS-95 to Destroy Bacterial Biofims

**Figure 5:**
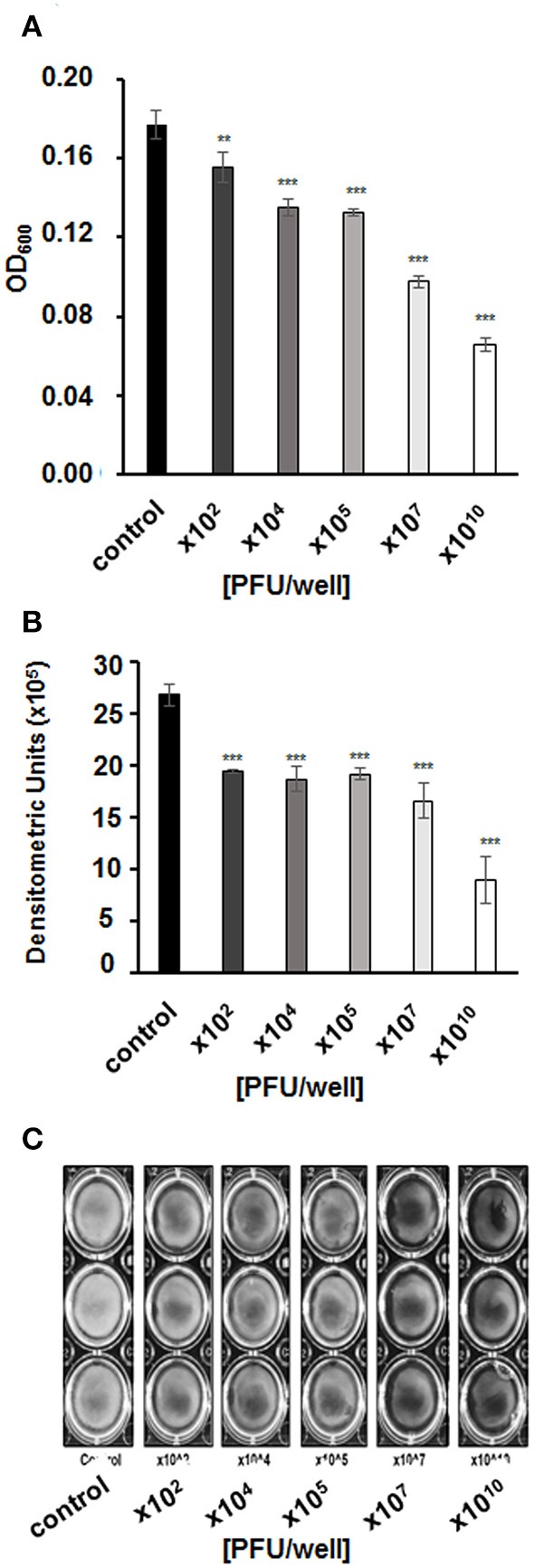
Biofilm density after a 4-h incubation with vB_EcoS-95 phage added to final titer of 10^2^,10^4^,10^5^,10^7^, or 10^10^ PFU/well. Results were **(A)** estimated by measuring the optical density at wave length 600 nm of the resuspended biofilms or **(B)** quantified densitometrically using Quantity One program from bacterial biofilms pictures **(C)**. Results are presented as mean values ± SD from three independent experiments. Statistical analyses were performed by *t*-test. The significance of differences between control (no infection) and particular variants of phage infection are observed and marked by asterisks *P* < 0.01 (^**^) or *P* < 0.001 (^***^).

**Figure 6:**
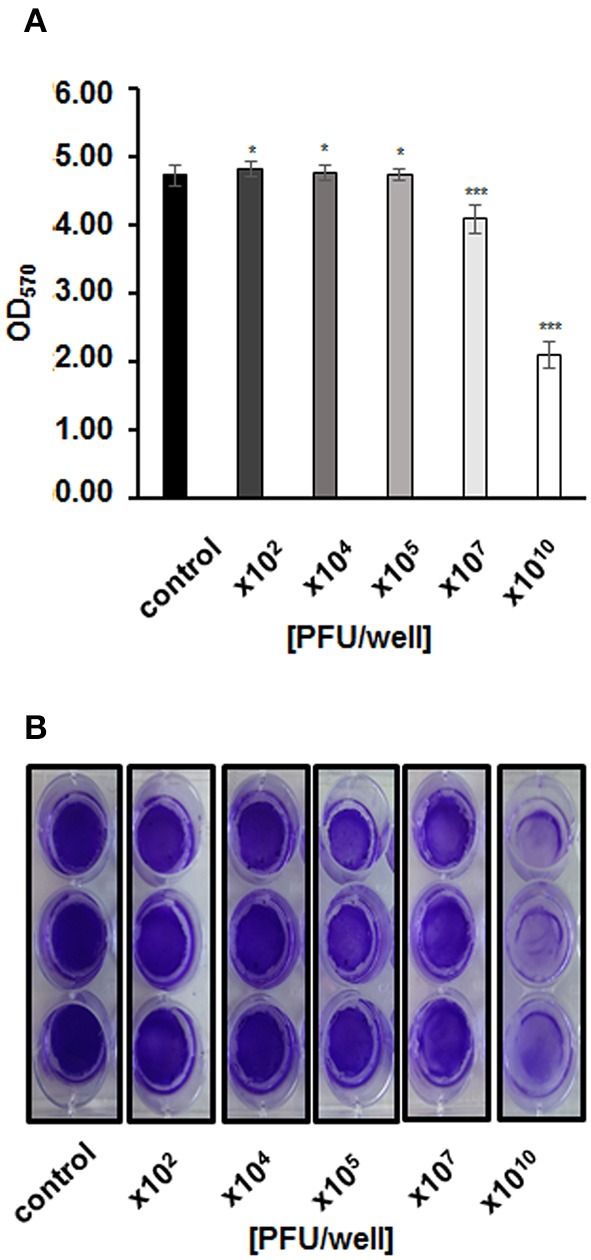
Biofilm biomass after infection with phage vB_EcoS-95, estimated by crystal violet staining method and **(A)** presented as optical density values measured at wave length 570 nm or **(B)** photographed. Phage was added to final titer of 10^2^,10^4^,10^5^,10^7^, or 10^10^ PFU/well and incubated with bacteria in biofilm for 4 h. Error bars indicate standard deviations of triplicate experiments. Statistical analysis (*t*-test) was performed between control and analyzed variants of phage infection. Statistical differences are marked by asterisks *P* < 0.05 (^*^) or *P* < 0.001 (^***^).

**Figure 7:**
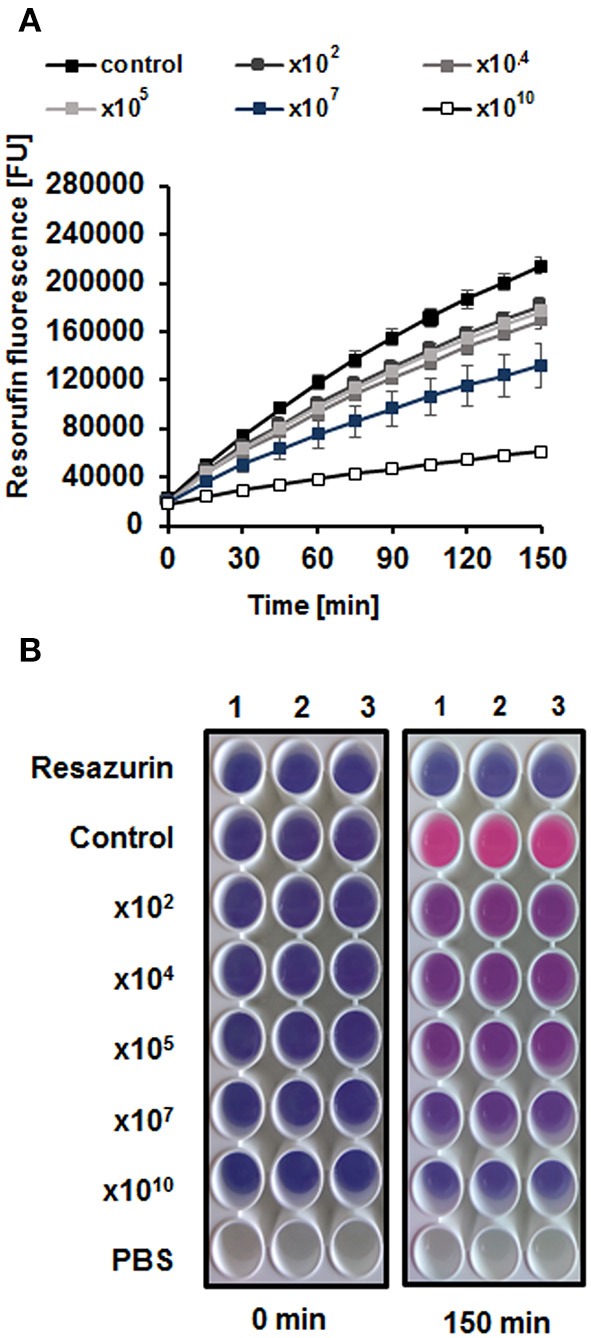
Metabolic activity / viability of cells in the biofilm, incubated for 4 h with different numbers of vB_EcoS-95 virions (10^2^,10^4^,10^5^,10^7^, or 10^10^ PFU/well), determined by resazurin assay in which the non-fluorescent blue resazurin is converted to the fluorescent pink resorufin. Bacterial cells viability is presented as fluorescence emitted by resorufin **(A)** and pink color of the bacterial culture in the wells **(B)**. Results are presented as mean values ± SD from three independent experiments. Please note that in some cases, the bars are smaller than sizes of symbols.

We asked whether vB-EcoS-95 is able to destroy biofilms formed by *E. coli* cells. Thus, we have tested biofilm density (by measuring the optical density at λ600 nm of the resuspended biofilm), biofilm biomass (using the crystal violet assay), and metabolic activity of cells in the biofilm (using the resazurin assay) after administration of different numbers of vB-EcoS-95 virions. In all these tests, we have observed deleterious effects of the tested bacteriophage on bacterial biofilm: its density (Figure 5), biomass (Figure 6), and metabolic activity (Figure 7). In all tests, the effects of the bacteriophage on the biofilm depended on number of virions used in the experiments, with the most pronounced effects at the highest number of administered virions. We are aware that the results obtained in various kinds of experiments differ in details. This is perhaps due to various sensitivities of different methods. Nevertheless, the general trend of the effects of phage vB-EcoS-95 on the bacterial biofilm is the same in all performed tests.

**Figure 8:**
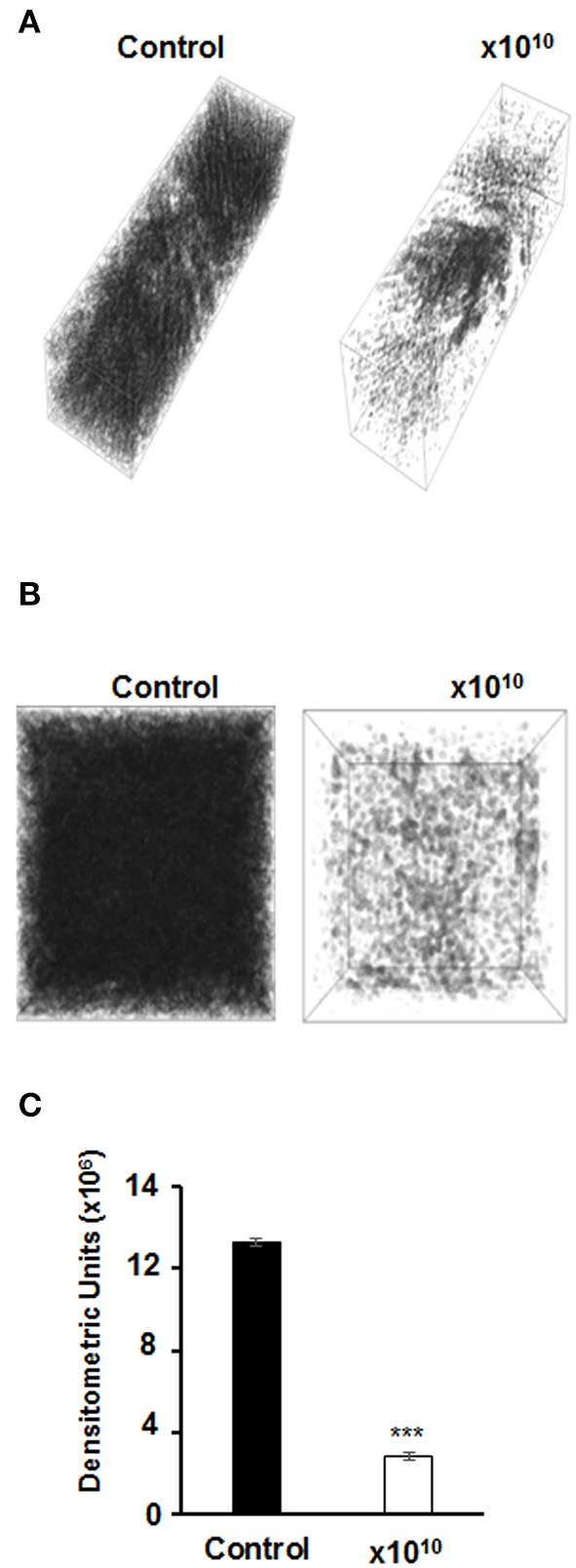
Images of bacterial biofilms untreated and treated for 4 h with vB_EcoS-95 phage lysate added to a final titer of 10^10^, performed by holographic 3D microscopy **(A,B)** and densitometry analysis of the obtained microscopic data **(C)**. Statistical analyses were performed by *t*-test. The significance of differences between compared experimental variants were marked by asterisks *P* < 0.001 (^***^).

The effects of vB-EcoS-95 on the biofilm destruction have been confirmed in experiments with holographic 3D microscopy. Significant lowering of the biofilm density could be observed after addition of phage vB-EcoS-95 lysate to *E. coli*-formed biofilm (Figure 8).

### Characterization of the vB-EcoS-95 Genome

**Figure 9:**
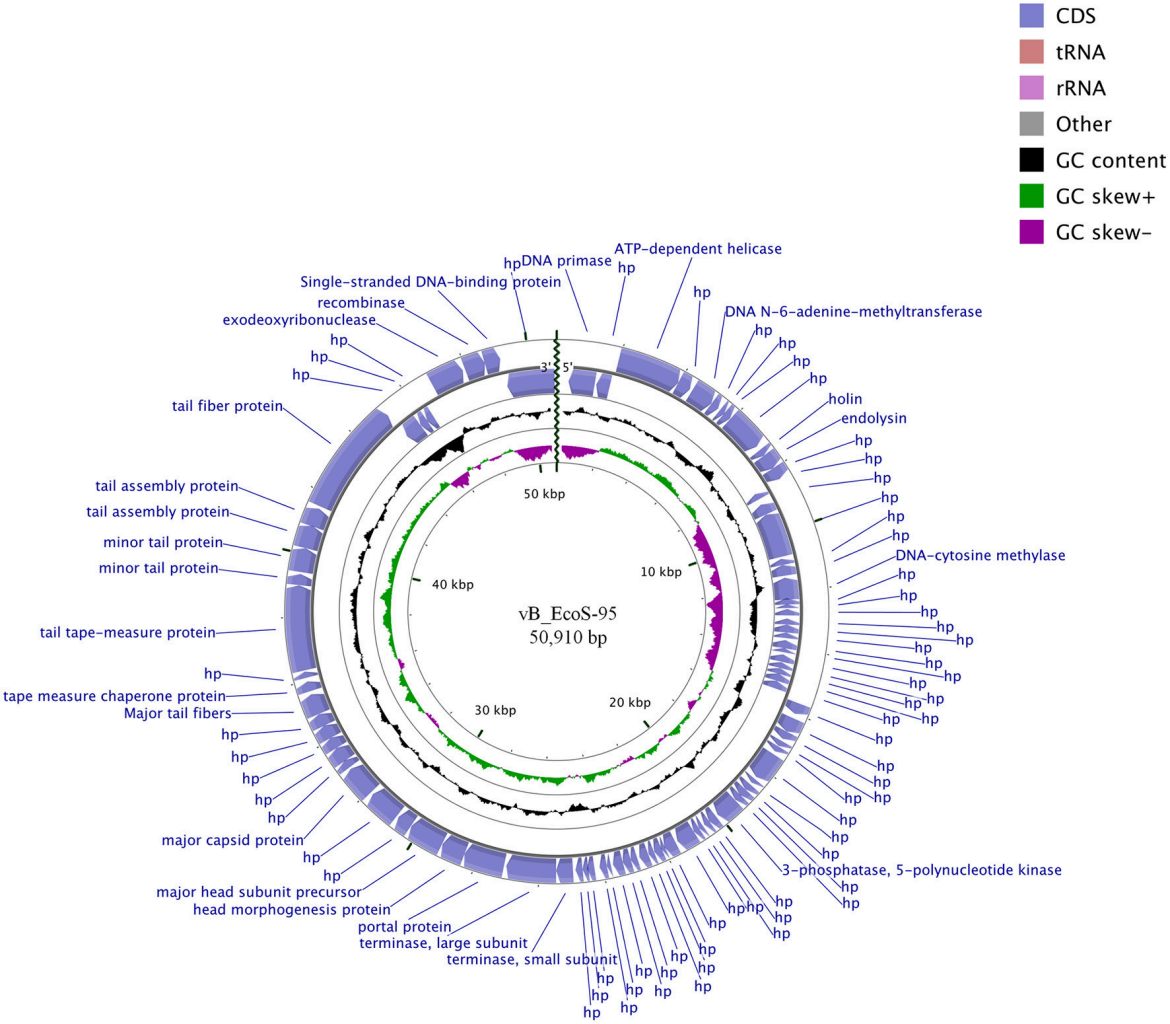
BRIG- and CGView-derived schematic map of circular genome of the vB_EcoS-95 phage. The inner rings show genome location, GC skew + (green) and—(purple) and GC content (black). Two the most external rings show identified open reading frames (blue arrows) and results of genome annotation process.

**Figure 10:**
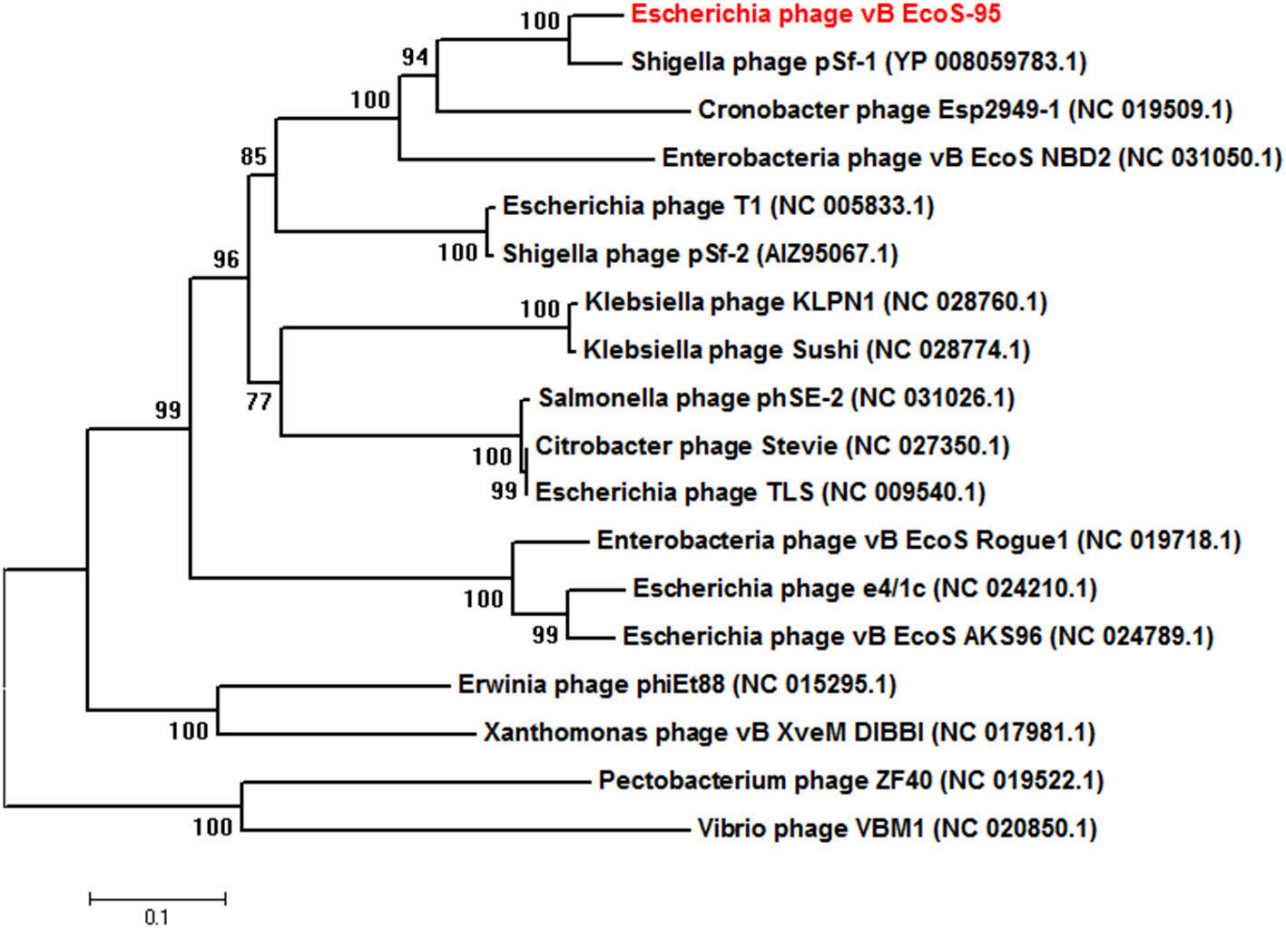
Neighbor-joining phylogenetic tree of the terminase large subunit (TerL) amino acid sequences showing the phylogenetic position of phage vB_EcoS-95 (in red color). The reference sequences were collected from the NCBI database. The tree was constructed using MEGA 7 after performing a sequence alignment using MUSCLE. Bootstrap values, calculated based on 1,000 resamplings, are shown at the nodes.

**Figure 11:**
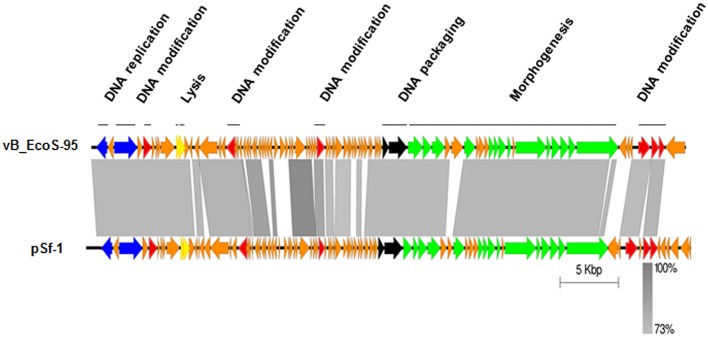
Easyfig output image of the genomic comparison between phage vB_EcoS-95 and the most related phage pSf-1. Phage genomes are presented by linear visualization with coding regions shown as arrows. Selected open reading frames are colored in relation to their functions. The percentage of sequence similarity is indicated by the intensity of the gray color. Vertical blocks between analyzed sequences indicate regions with at least 73% of similarity.

The whole genome of vB-EcoS-95 has been sequenced. It consists of 50,910 bp and is deposited in GenBank (accession number: MF564201). The map of this genome is presented in Figure 9. Analysis of the vB-EcoS-95 genome indicated that it is a double-stranded DNA, with a 45% total G+C content. This analysis has shown 45 putative promoters, 30 putative transcription terminators and 89 open reading frames (ORFs). Among all identified coding regions, only 24 ORFs were predicted to be functional genes. As showed in Figure 10, the large terminase subunit of vB-EcoS-95 bacteriophage presents the highest identity with large terminase subunit of other virulent *Siphoviridae* phage, pSf-1, that infects *Shigella flexneri* (Jun et al., 2013). Sequence similarity searches revealed that vB-EcoS-95 presents ~74% nucleotide sequence identity with phage pSf-1. Besides, these two phages are highly similar in gene inventory. As indicated in Figure 11, both phage genomes contain blocks of genes in which genes are clustered by function and encode products that operate in similar way, such as proteins responsible for DNA replication, modification, recombination etc. Interestingly, in both cases, genes coding for products participating in DNA packaging and morphogenesis are located at the middle and the end of the genome.

### Mass-Spectrometric Identification of Phage Proteins

The proteins of phage vB-EcoS-95 detected by MS are listed in Table 6 with their observed molecular mass, the number of identified peptides in each protein and the corresponding protein sequence coverage. For all proteins, the identification was based on fragmentation patterns of unique peptides. The data analysis allowed us to assign 16 vB-EcoS-95 phage proteins to annotated open reading frames (ORFs). In this manner, protein annotation of 10 *in silico*-predicted structural proteins was confirmed. In addition, 6 gene products having little or no similarity to known phage proteins could be classified as proteins of unknown function. The identification analyses of the proteins can be verified in the Supplementary Tables of data available with the online version of this paper (Supplementary Material – MS identification data).

**Table 6:** Mass spectrometry analysis of bacteriophage vB_EcoS-95 virion.

**Detected protein**	**Predicted function**	**Molecular mass (kDa)**	**Number of peptides**	**Sequence coverage (%)**	**Protein Score**
vB_EcoS-95_0064	Portal protein	48.5	12	40.7	127.57
vB_EcoS-95_0065	Head morphogenesis protein	28.7	12	43.43	57.75
vB_EcoS-95_0066	Major head subunit precursor	40.5	5	20.49	1.73
vB_EcoS-95_0067	Unknown protein	17.1	7	55.35	83.22
vB_EcoS-95_0068	Unknown protein	34.9	12	46.29	693.13
vB_EcoS-95_0069	Major capsid protein	36.0	17	59.44	815.89
vB_EcoS-95_0073	Unknown protein	16.3	2	13.61	3.83
vB_EcoS-95_0075	Major tail fibers	24.4	8	38.18	195.94
vB_EcoS-95_0078	Tail tape-measure protein	98.1	56	53.30	1479.50
vB_EcoS-95_0079	Minor tail protein	12.9	2	24.14	15.08
vB_EcoS-95_0080	Minor tail protein	28.4	5	25.69	3.41
vB_EcoS-95_0082	Tail assembly protein	20.7	2	8.59	99.97
vB_EcoS-95_0083	Tail fiber protein	132.4	44	42.70	1095.31
vB_EcoS-95_0084	Unknown protein	22.0	2	13.24	24.36
vB_EcoS-95_0085	Unknown protein	10.0	3	55.43	9.69
vB_EcoS-95_0090	Unknown protein	60.1	22	43.65	910.97

## Discussion

A newly isolated bacteriophage, vB-EcoS-95, infecting *E. coli* strains, has been characterized. It infects various *E. coli* strains, including those lysogenized with different lambdoid phages and clinical isolates, however, *E. coli* O157:H7 bacteria are resistant to this phage.

The most intriguing property of this phage is its extremely rapid development in *E. coli* cells. Under standard laboratory conditions, i.e., growth of host bacteria at 37°C in LB medium, the latent period was as short as 4 min, and the lysis of host culture was complete within 20-25 min. This was accompanied with relative high average burst size of 115 pfu/cell. For comparison, one-step growth experiment of phage pSf-1 (the most closely related phage to vB-EcoS-95, reported to date; however, note that the level of similarity of pSf-1 and vB-EcoS-95 genomes, 74%, is still moderate) and other closely related phage pSf-2 showed that the latent period was longer (10 and 30 min, respectively) and burst size was lower (around 87 and 16 PFU/cell, respectively) (Jun et al., 2013, 2016). Another interesting feature of this phage is its ability to destroy bacterial biofilm which was demonstrated by using various methods, including crystal violet and resazurin assays, as well as holographic 3D microscopy.

Genome of vB-EcoS-95 revealed 74% similarity to previously described phage pSf-1, infecting Shigella *flexneri* (Jun et al., 2013). However, unlike that phage, vB-EcoS-95 does not infect *S. flexneri*. In fact, only some *E. coli* strains could be effectively infected by the newly isolated phage. Moreover, contrary to pSf-1, phage vB-EcoS-95 reveals an extremely short latent period (about 4 min) after infection of the *E. coli* host (see above). It is unclear what causes such a rapid lytic development of vB-EcoS-95. Genome analysis indicated that this phage does not encode its own DNA polymerase, therefore, it is unlikely that extremely quick phage DNA synthesis is responsible for this phenomenon, though one cannot exclude that initiation of this process is particularly efficient. On the other hand, it appears that vB-EcoS-95 encodes an untypical lytic protein (Supplementary Material—genome annotation) which might potentially contribute to rapid lysis of the host cell.

All the properties of vB-EcoS-95 make it a potentially attractive phage in further studies on its use in biotechnological applications and/or as a factor for food protection or therapeutic agent (in phage therapy). Particularly, very rapid lytic development, accompanied with a relatively high burst size indicate that this phage can destroy host cells very rapidly, which is beneficial in both phage therapy and protection of food or various materials against bacterial colonization. Effective destruction of bacterial biofilm may be of particular importance, as formation of such a structure by bacteria protects them against various antibacterial agents, including antibiotics. On the other hand, vB-EcoS-95 effectively infects only some *E. coli* strains, including some clinical isolates, but excluding *E. coli* O157:H7. This might be a potential limitation in the medical use of vB-EcoS-95, though one must note that specificity of phages to certain strains is a commonly occurring feature of these viruses. The vB-EcoS-95 phage survives well at relatively low temperatures, but <50% virions could retain infectivity at 40°C. This might suggest a potential limitation in phage therapy, as bacterial infections usually cause fever in patients. On the other hand, in phage therapy procedures, a large excess of bacteriophages is usually applied, therefore, such a survival rate should not significantly influence efficacy of the therapy. Finally, although appearance of bacteria resistant to vB-EcoS-95 was as frequent as appearance of bacteria resistant to antibiotic, survival of *E. coli* cells after administration of the phage was significantly lower than that after administration of rifampicin. This suggest an advantage of the use of vB-EcoS-95 in phage therapy or protection of food or various materials.

In summary, the newly isolated vB-EcoS-95 phage, infecting *E. coli* cells, reveals various specific features, particularly, rapid development and cell lysis, ability to destroy bacterial biofilm, and untypical lytic protein, which suggest that further studies on its use in biotechnological and/or medical applications are desired.

## Data Availability Statement

The genome sequence is available at GenBank (accession number: MF564201). Raw data are available from authors on request.

## Author Contributions

GT performed most of the experiments on characterization of bacteriophage development and its ability to destroy bacterial biofilms, participated in data analysis and helped to draft methodology chapter. SB designed the experimental work, helped to perform some of the experiments and to analyse obtained data. BN-F participated in the planning of experiments and genomic analyses, helped in data analysis and drafting the manuscript. TG analyzed sequence of phage genome. AJ-K isolated vB_EcoS-95 phage from urban sewage sample and participated in genomic analyses. AN and AD performed a part of experimental work on phage biology. MR performed electron microscopic analyses of the phage. GW participated in analysis of the results and drafting the manuscript. AW was the principal investigator of the project, supervised the work, and participated in drafting the manuscript.

### Conflict of Interest Statement

The authors declare that the research was conducted in the absence of any commercial or financial relationships that could be construed as a potential conflict of interest.

## Supplementary Material

Table S1 Bacteriophage vB_EcoM-95 genome annotations.

Table S2 Mass spectrometry analysis of bacteriophage vB_EcoS-95 virion.
